# Microglia-Derived Olfactomedin-like 3 Is a Potent Angiogenic Factor in Primary Mouse Brain Endothelial Cells: A Novel Target for Glioblastoma

**DOI:** 10.3390/ijms232314613

**Published:** 2022-11-23

**Authors:** Laila M. Joseph, Ryan G. Toedebusch, Eshetu Debebe, Aurelie H. Bastian, Christopher A. Lucchesi, Shafee Syed-Quadri, Luke A. Wittenburg, Xinbin Chen, Frederick J. Meyers, Christine M. Toedebusch

**Affiliations:** 1 Department of Surgical and Radiological Sciences, School of Veterinary Medicine, University of California, Davis, CA 95616, USA; 2 Comprehensive Cancer Center, Davis School of Medicine, University of California, Sacramento, CA 95817, USA; 3 Department of Internal Medicine, Division of Hematology and Oncology, Center for Precision Medicine, Davis School of Medicine, University of California, Sacramento, CA 95817, USA

**Keywords:** angiogenesis, glioblastoma, microglia, cancer, central nervous system, C57Bl/6 mice

## Abstract

Neoangiogenesis, a hallmark feature of all malignancies, is robust in glioblastoma (GBM). Vascular endothelial growth factor (VEGF) has long been regarded as the primary pro-angiogenic molecule in GBM. However, anti-VEGF therapies have had little clinical efficacy, highlighting the need to explore VEGF-independent mechanisms of neoangiogenesis. Olfactomedin-like 3 (OLFML3), a secreted glycoprotein, is an established proangiogenic factor in many cancers, but its role in GBM neoangiogenesis is unknown. To gain insight into the role of OLFML3 in microglia-mediated angiogenesis, we assessed endothelial cell (EC) viability, migration and differentiation following (1) siRNA knockdown targeting endogenous EC *Olfml3* and (2) EC exposure to human recombinant OLFML3 (rhOLFML3; 10 ng/mL, 48 h), and conditioned medium (CM) from isogenic control and *Olfml3^−/−^* microglia (48 h). Despite a 70% reduction in *Olfml3* mRNA levels, EC angiogenic parameters were not affected. However, exposure to both rhOLFML3 and isogenic control microglial CM increased EC viability (*p* < 0.01), migration (*p* < 0.05) and differentiation (*p* < 0.05). Strikingly, these increases were abolished, or markedly attenuated, following exposure to *Olfml3^−/−^* microglial CM despite corresponding increased microglial secretion of VEGF-A (*p* < 0.0001). Consistent with reports in non-CNS malignancies, we have demonstrated that OLFML3, specifically microglia-derived OLFML3, promotes VEGF-independent angiogenesis in primary brain microvascular ECs and may provide a complementary target to mitigate neovascularization in GBM.

## 1. Introduction

In 2000, Hanahan and Weinberg proposed neoangiogenesis as one of six hallmark features of cancer [1,2]. While malignant cells were initially identified as the pro-angiogenic source, the tumor microenvironment (e.g., immune cells) has become increasingly recognized for its role in promoting neovascularization [3,4]. Neoangiogenesis is particularly robust in glioblastoma (GBM) [5,6], whereby sprouting angiogenesis and microvascular proliferation promote the rapid growth of this uniformly lethal brain tumor. 

While vascular endothelial growth factor (VEGF) is the most abundant angiogenic factor in GBM [7], tumor recurrence and neoangiogenesis observed following anti-VEGF therapy strongly suggests VEGF-independent mechanisms of angiogenesis in GBM. Moreover, targeting VEGF minimally improved progression-free survival in clinical patients [8], highlighting the complexity of angiogenesis in GBM. Indeed, hypoxia-dependent and independent mechanisms have been identified across multiple cell types [9,10,11]. Glioma-associated microglia (GAM) infiltrate GBM and play a critical role in the induction of angiogenesis [12,13]. Covering as much as 20% of the intratumoral vasculature [13], microglia have intimate contact with endothelial cells (ECs). Moreover, tumor-derived transforming growth factor-beta (TGFβ), a key pro-tumorigenic cytokine, polarizes microglia to support vascular development through the secretion of several pro-angiogenic molecules [14,15]. 

Olfactomedin-like 3 (OLFML3), a secreted glycoprotein, is an emerging pro-tumorigenic molecule in non-central nervous system (CNS) cancer. OLFML3 is a top-five differentially expressed protein in the stroma of human colon cancer and is recognized as a disease biomarker [16]. Moreover, transcriptional repression of *Olfml3* by the Breast Carcinoma Metastasis Suppressor gene inhibited cell migration and invasion in breast cancer cell lines [17], indicating its relevance in epithelial-to-mesenchymal transition and metastasis. OLFML3 has also been directly implicated in angiogenesis. In an elegant study by Imhof and colleagues [18], OLFML3 was shown to be necessary for pericyte-mediated vascular development in the developing mouse pup. Within the context of cancer, increased OLFML3 immunostaining has been documented in the tumor-infiltrating vasculature of colorectal, uterine, lung and prostate carcinoma [19]. Importantly, targeting OLFML3 has been linked with improved outcomes in preclinical cancer models. Antibody-mediated inhibition of OLFML3, as well as genetic silencing of *Olfml3,* resulted in reduced tumor vascularization and growth, which ultimately prolonged survival in mouse models of lung and colorectal cancer [19,20]. 

We have recently demonstrated that OLFML3 is a relevant pro-tumorigenic molecule in GBM. Indeed, *OLFML3* mRNA expression is positively correlated with glioma grade, with the highest expression observed in adult GBM [21]. While many cell types within the murine CNS express *Olfml3*, including microglia [22], endothelial cells (ECs) [23] and glioma cells [21,24,25], the role of OLFML3 in the healthy CNS remains incompletely characterized. We have demonstrated that microglia-derived OLFML3 promotes pro-tumorigenic microglia function and glioma cell malignancy [21]. However, the pro-angiogenic role of OLFML3 in GBM has not been explored. In a healthy brain, ECs express low to moderate levels of *Olfml3*. However, expression is dramatically increased in encephalopathies characterized by robust neovascularization [26,27,28], including stroke, epilepsy and encephalitis [23]. Thus, EC-derived *Olfml3* is likely relevant in CNS neovascularization. 

Together, our recent finding that microglia-derived OLFML3 promotes malignant features of glioma cells, coupled with the general angiogenic effects of OLFML3, prompt us to speculate that microglia-derived OLFML3 may also promote EC neoangiogenesis in GBM. To date, neither the role of endogenous *Olfml3*, nor the effect of microglia-derived OLFML3, on mouse brain EC angiogenesis have been described. Therefore, this study aimed to test the hypotheses that: (1) endogenous *Olfml3* is necessary and (2) microglia-derived OLFML3 is sufficient to promote angiogenesis in mouse primary brain microvascular ECs. Utilizing primary mouse brain ECs, angiogenic parameters were assessed following (a) siRNA knockdown of *Olfml3,* (b) exposure to recombinant human OLFML3 (rhOLFML3), and c) exposure to conditioned medium (CM) from isogenic control and *Olfml3* null (*Olfml3^−/−^*) microglia.

## 2. Results

### 2.1. Knockdown of Endogenous Olfml3 Does Not Alter Primary Mouse Brain EC Function

**Figure 1:**
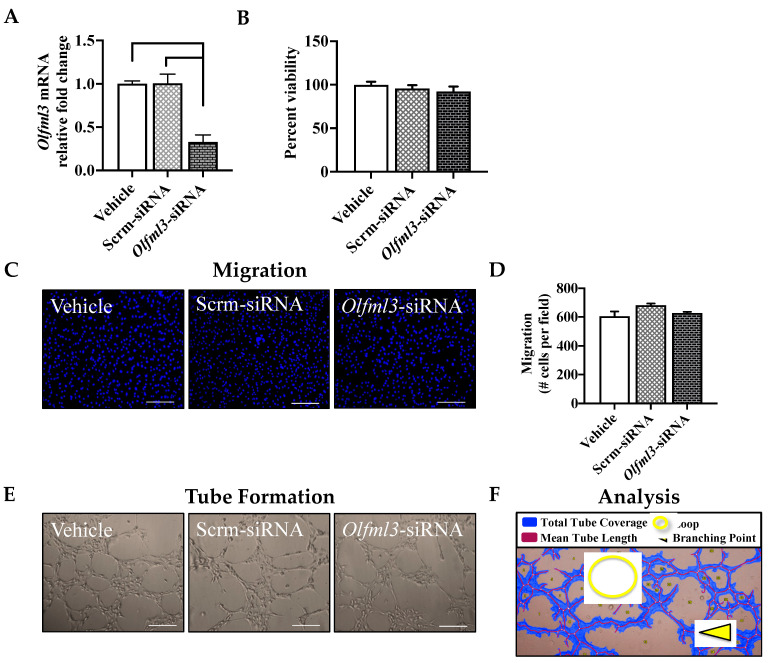

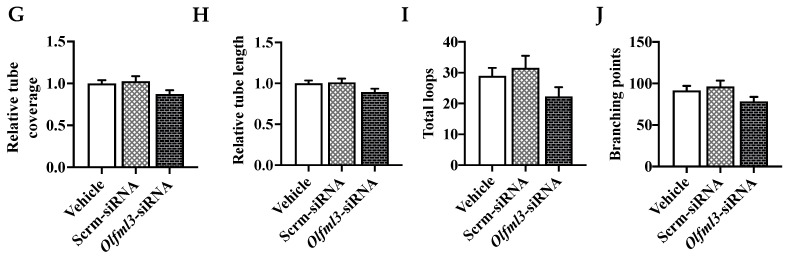
Reduced endogenous *Olfml3* mRNA levels do not affect primary mouse brain endothelial cell (EC) angiogenesis. (**A**) Transfection with siRNA targeting *Olfml3* (*Olfml3*-siRNA) reduced EC *Olfml3* mRNA expression by >60% relative to vehicle-and non-targeting siRNA-treated (scrm-siRNA) ECs. (**B**) Reduced mRNA levels of *Olfml3* did not alter EC viability (*p* = 0.7773). (**C**) Representative images of primary mouse brain EC migration following vehicle treatment and transfection with scrm-siRNA and *Olfml3*-siRNA; scale bars 50 μm. (**D**) Reduced mRNA levels of *Olfml3* did not alter EC migration (*p* = 0.1154). (**E**) Representative images of primary mouse brain EC differentiation across conditions; scale bars 50 μm. (**F**) Representative image of Wimasis analysis of key parameters of EC differentiation. EC differentiation was not altered by reduced *Olfml3* mRNA levels, as determined by (**G**) relative tube coverage (*p* = 0.1130), (**H**) relative tube length (*p* = 0.1431), (**I**) total loops (*p* = 0.1687), and (**J**) branching points (*p* = 0.1788). Comparisons based on one-way ANOVA with Tukey’s multiple comparisons test; bars represent group mean with standard error of the mean (SEM).

To evaluate the endogenous role of *Olfml3* in primary mouse brain EC angiogenesis, we transfected ECs with *Olfml3*-siRNA, which resulted in a marked knockdown of *Olfml3* mRNA levels compared to vehicle- (*p* = 0.0192) and non-targeting siRNA-treated (scrm-siRNA; *p* = 0.0188) ECs (Figure 1A). Reduced *Olfml3* mRNA levels did not alter EC viability compared to vehicle- (*p* = 0.4461) or scrm-siRNA-treated (*p* = 0.8383) ECs (Figure 1B). Similarly, migration was not affected by *Olfml3*-siRNA compared to vehicle- (*p* = 0.7663) or scrm-siRNA-treated (*p* = 0.2635) ECs (Figure 1C,D). Lastly, EC differentiation was not affected by reduced *Olfml3* mRNA levels, as assessed by several parameters, including relative tube coverage, relative tube length, total loops, and branching points (Figure 1E–J). While we did not eliminate EC *Olfml3* expression, these findings indicate that reduced levels of endogenous *Olfml3* do not affect viability, migration nor differentiation in juvenile C57Bl/6 brain microvascular ECs.

### 2.2. Exogenous OLFML3 Promotes Primary Mouse Brain EC Viability, Migration, and Proliferation

**Figure 2:**
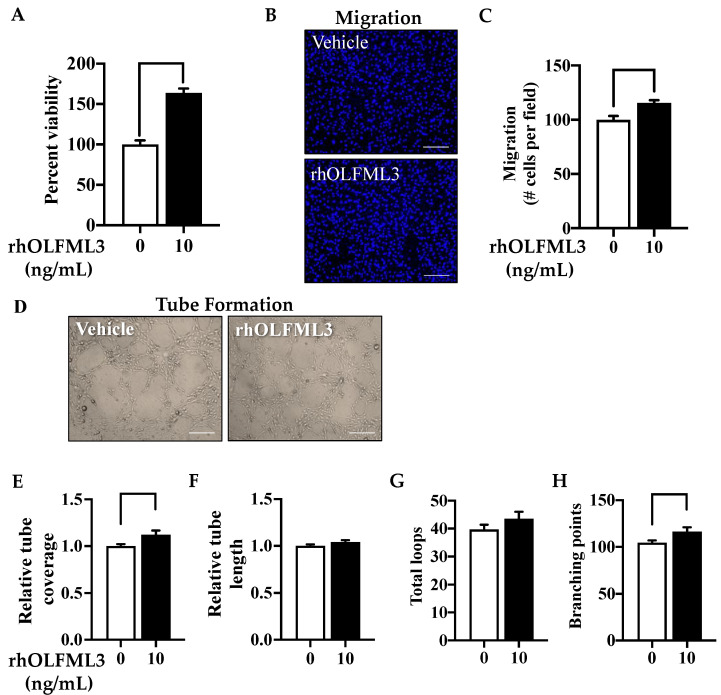
OLFML3 is sufficient to induce primary mouse brain EC angiogenesis. (**A**) Exposure to recombinant human OLFML3 (rhOLFML3; 10 ng/mL, 48 h) increased mouse brain endothelial cell (EC) viability relative to vehicle-treated cells. (**B**) Representative images of primary mouse brain EC migration following exposure to vehicle and rhOLFML3 (10 ng/mL; 48 h); scale bars 50 µm. (**C**) EC migration was increased following exposure to rhOLFML3 (10 ng/mL, 48 h). (**D**) Representative images of primary mouse brain EC differentiation following exposure to vehicle and rhOLFML3 (10 ng/mL; 48 h); scale bars 50 µm. Exposure to rhOLFML3 (10 ng/mL, 48 h) increased EC (**E**) tube coverage and (**H**) branching points relative to vehicle-treated cells. Comparisons based on unpaired student’s t test; bars represent group mean with standard error of the mean (SEM).

We next sought to determine the effect of exogenous OLFML3 on EC angiogenesis. To directly evaluate this, we treated primary mouse brain ECs with recombinant human OLFML3 (rhOLFML3; 10 ng/mL, 48 h). Exposure to rhOLFML3 nearly doubled EC viability relative to vehicle-treated ECs (*p* = 0.0001) (Figure 2A). Migration was also increased following rhOLFML3 treatment (*p* = 0.02) (Figure 2B,C). Moreover, multiple parameters of differentiation were increased by rhOLFML3 exposure. Relative tube coverage (*p* = 0.0346) (Figure 2E) and branching points (*p* = 0.0410) (Figure 2H) were both increased following exposure to rhOLFML3. Neither relative tube length (*p* = 0.1381) (Figure 2F), nor total loops (*p* = 0.2147) (Figure 2G) were altered by rhOLFML3 treatment. These findings support our hypothesis that OLFML3 is sufficient to promote angiogenesis in brain microvascular ECs. 

**Figure 3:**
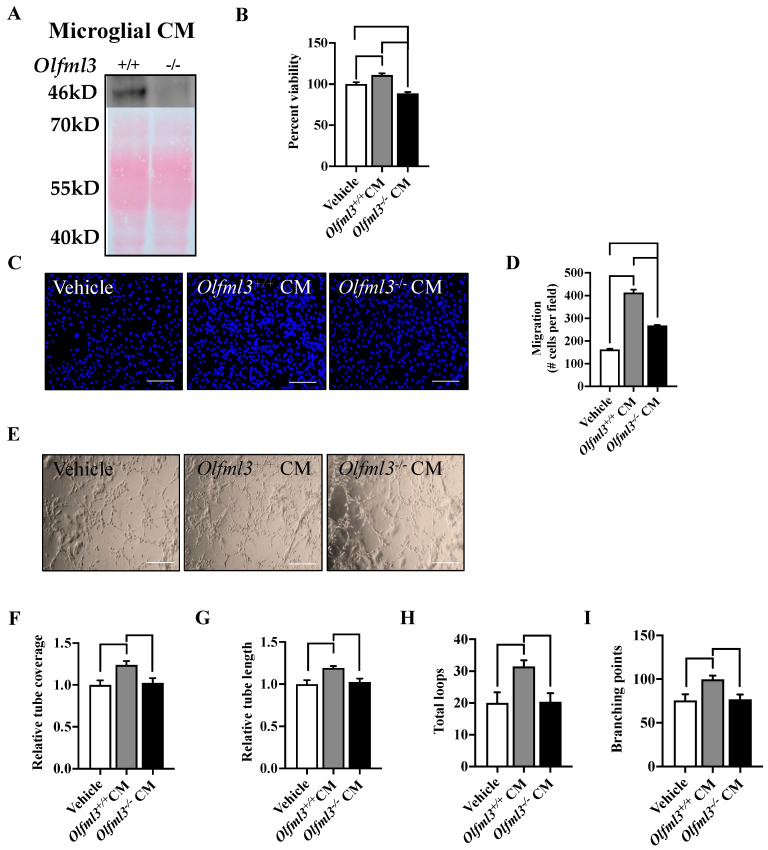
Microglia-derived OLFML3 is necessary for microglia-induced angiogenesis. (**A**) Representative immunoblot for OLFML3 on conditioned medium (CM) revealed positivity immunoreactivity at the predicted molecular weight (46 kD) in isogenic control medium, but not CM from *Olfml3^−/−^* microglia. The corresponding membrane Ponceau stain is pictured as a loading control. (**B**) Exposure to isogenic control CM increased EC viability. Notably, EC viability was reduced following exposure to *Olfml3^−/−^* microglial CM relative to vehicle-treated and isogenic control CM. (**C**) Representative images of primary mouse brain EC migration across experimental conditions. (**D**) Exposure to isogenic control and *Olfml3^−/−^* microglial CM increased EC migration. However, EC migration was mitigated following exposure to *Olfml3^−/−^* microglial CM relative to isogenic control CM. (**E**) Representative images of primary mouse brain EC differentiation across experimental conditions; scale bars 50 µm. EC differentiation was increased following exposure to isogenic control CM, but not *Olfml3^−/−^* microglial CM, as determined by (**F**) relative tube coverage, (**G**) relative tube length, (**H**) total loops, and (**I**) branching points. Comparisons based on one-way ANOVA with Tukey’s multiple comparisons test; bars represent group mean with standard error of the mean (SEM).

As rhOLFML3 was sufficient to induce several phases of angiogenesis in primary mouse brain ECs, we further explored the contribution of microglia-derived OLFML3 on EC angiogenesis through exposure to conditioned medium (CM; 48 h) from cultured isogenic control (*Olfml3^+/+^)* and *Olfml3* null (*Olfml3^−/−^)* microglia. OLFML3 was not detectable in *Olfml3^−/−^* CM (Figure 3A). Exposure to *Olfml3^+/+^* CM increased EC viability relative to vehicle-treated cells (*p* = 0.0033) (Figure 3B). However, the absence of OLFML3 (*Olfml3^−/−^* CM) significantly reduced EC viability compared to vehicle-treated (*p* = 0.0018) and *Olfml3^+/+^* CM-treated (*p* < 0.0001) ECs (Figure 3B). Moreover, *Olfml3^+/+^* CM treatment increased EC migration compared to vehicle treatment (*p* < 0.0001) (Figure 3C,D). EC migration was also increased following exposure to *Olfml3^−/−^* CM (268.1 ± 3.2; *p* < 0.0001), but the number of migrated cells was markedly attenuated compared to *Olfml3^+/+^* CM-treated ECs (*p* < 0.0001) (Figure 3C,D). EC differentiation, while increased following exposure to *Olfml3^+/+^* CM, was not altered by exposure to Olfml3^−/−^ CM.

Relative tube coverage (Figure 3E,F), relative tube length (Figure 3E,G), total loops (Figure 3E,H), and branching points (Figure 3E,I) were all increased in *Olfml3^+/+^* CM-treated ECs relative vehicle- and *Olfml3^−/−^* CM-treated ECs, respectively (*p* < 0.05). These findings suggest that microglia-derived OLFML3 is sufficient to promote all parameters of angiogenesis and may be necessary for EC viability and differentiation in juvenile C57Bl/6 brain microvascular ECs. 

### 2.3. Loss of Microglial Olfml3 Increases Expression of Alternate Microglia-Derived Pro-Angiogenic Molecules

**Figure 4:**
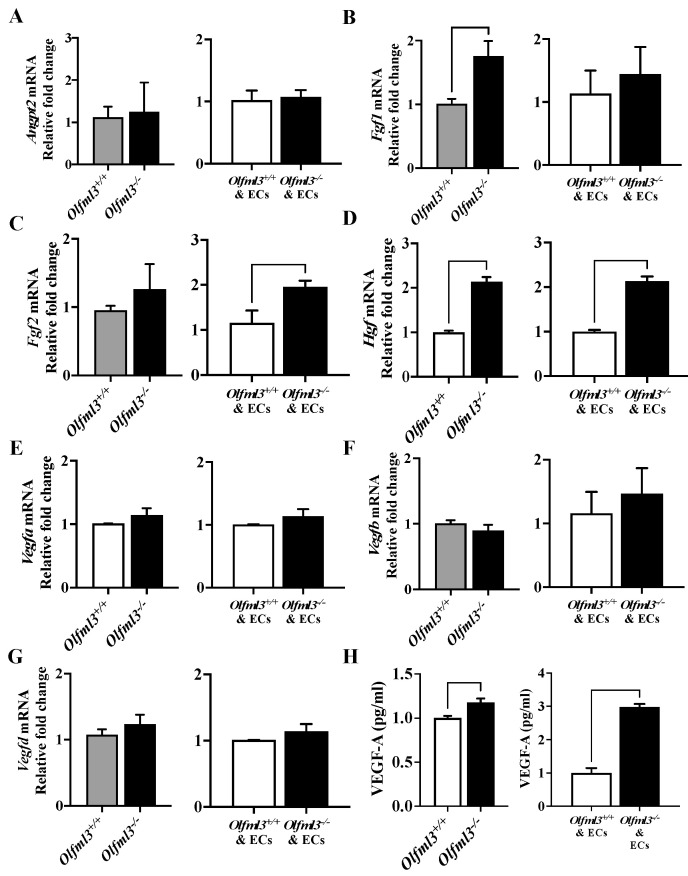
Loss of microglial *Olfml3* increased expression of alternate microglia-derived pro-angiogenic factors. (**A**) mRNA levels of *Angpt2* were not affected by loss of microglial *Olfml3* when cultured alone (*p* = 0.8582) or in the presence of ECs (48 h; *p* = 0.7947). (**B**) *Fgf1* mRNA was increased in *Olfml3^−/−^* microglia cultured alone (*p* = 0.0243) but not in the presence of ECs (*p* = 0.5997). (**C**) *Fgf2* mRNA levels were not affected by loss of *Olfml3* in microglia cultured alone (*p* = 0.5139) but were moderately increased in the presence of ECs (*p* = 0.0407). (**D**) *Hgf* mRNA increased in *Olfml3^−/−^* microglia cultured alone (*p* = 0.0143) and in the presence of ECs. Notably, mRNA levels of VEGF family genes (**E**) *Vegfa*, (**F**) *Vegfb*, and (**G**) *Vegfd* were unchanged in *Olfml3^−/−^* microglia cultured alone or in the presence of ECs. (**H**) VEGF-A concentration, expressed as relative to isogenic control, in microglial supernatant was increased in *Olfml3^−/−^* microglia cultured alone and in the presence of ECs. Comparisons based on unpaired student’s t test; bars represent group mean with standard error of the mean (SEM).

We have previously demonstrated that loss of Olfml3-altered microglial expression of several pro-tumorigenic genes [21]. Given the marked attenuation of angiogenic parameters observed following EC exposure to *Olfml3^−/−^* microglia CM, we evaluated gene expression of several known pro-angiogenic molecules in isogenic control and *Olfml3^−/−^* microglia. Moreover, we evaluated mRNA levels of these same genes in microglia co-cultured with ECs to determine if loss of Olfml3 affected pro-angiogenic paracrine signaling between cell types. Microglial mRNA expression of pro-angiogenic angiopoietin-2 (Angpt2) was not affected by loss of Olfml3 in microglia cultured alone (*p* = 0.8582) or in the presence of ECs (*p* = 0.7947) (Figure 4A). However, fibroblast growth factor 1 (Fgf1) was moderately increased in *Olfml3^−/−^* microglia cultured alone (*p* = 0.0243), but not in the presence of ECs (*p* = 0.5997) (Figure 4B). Conversely, microglial fibroblast growth factor 2 (Fgf2) mRNA levels were not affected by loss of *Olfml3* in microglia cultured alone (*p* = 0.5139) but were moderately increased in the presence of ECs (*p* = 0.0407) (Figure 4C). The most striking change in gene expression observed was the increase in hepatocyte growth factor (Hgf) mRNA in *Olfml3^−/−^* microglia cultured alone (*p* = 0.0143) and in the presence of ECs (*p* < 0.0001) (Figure 4D). Notably, mRNA levels of VEGF family genes (Vegfa, Vegfb, Vegfd; Figure 4E–G) were not altered by the loss of *Olfml3* cultured alone or in the presence of ECs. mRNA levels of Vegfc were considerably lower in isogenic control and *Olfml3^−/−^* microglia, resulting in equivocal quantification and comparison (threshold cycle > 40); therefore, these data were not included. Unexpectedly, the quantification of VEGF-A in microglial supernatant demonstrated increased secretion of VEGF-A from *Olfml3^−/−^* microglia cultured alone (*p* = 0.0160) and in the presence of ECs (*p* < 0.0001) (Figure 4H). Despite increases in several pro-angiogenic factors, including secreted VEGF-A, all angiogeneic parameters were reduced in ECs following exposure to *Olfml3^−/−^* microglia CM. Collectively, these experiments implicate microglial OLFML3 as a potent angiogenic stimulus.

### 2.4. Pro-Angiogenic Gene Expression in Brain ECs Is Minimally Influenced by the Absence of Microglial Olfml3 

**Figure 5:**
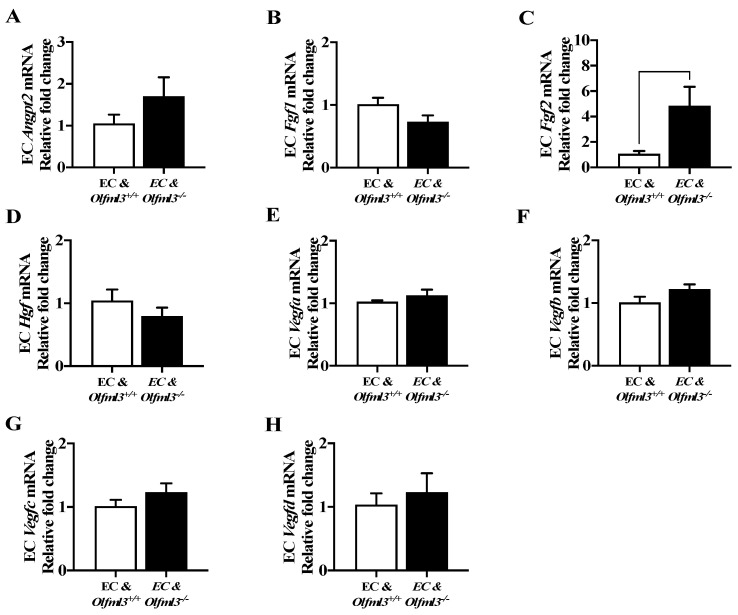
Brain EC expression of most pro-angiogenic genes is not influenced by the absence of microglial *Olfml3.* mRNA levels of (**A**) *Angpt2* (*p* = 0.2619), (**B**) *Fgf1* (*p* = 0.1174), (**D**) *Hgf*, (**E**) *Vegfa* (*p* = 0.3186), (**F**) *Vegfb* (*p* = 0.1311), (**G**) *Vegfc* (*p* = 0.2455)*,* and (**H**) *Vegfd* (*p* = 0.6027) were unchanged in ECs co-cultured with isogenic control and *Olfml3^−/−^* microglia. (**C**) mRNA levels of *Fgf2* were increased in ECs co-cultured with *Olfml3^−/−^* microglia relative to ECs co-cultured with isogenic control microglia. Comparisons based on unpaired student’s t test; bars represent group mean with standard error of the mean (SEM).

To determine whether *Olfml3^−/−^* microglia induced changes in EC gene expression, we evaluated the mRNA levels of pro-angiogenic molecules in ECs following co-culture with microglia. The loss of microglial *Olfml3* induced an increase in EC *Fgf2* expression (*p* = 0.0485; Figure 5C). All remaining genes assayed were not altered by the loss of microglial *Olfml3* (Figure 5), suggesting that alterations to EC gene expression were not contributing to the reduction in angiogenic parameters observed following treatment with *Olfml3^−/−^* microglial CM. 

## 3. Discussion

We have demonstrated that OLFML3 is a potent inducer of angiogenesis in brain microvascular ECs. While reduced endogenous levels of *Olfml3* did not perturb angiogenesis, exogenous OLFML3 was sufficient to induce multiple parameters of angiogenesis in brain microvascular ECs. Microglia-derived OLFML3 significantly contributed to increased EC angiogenic parameters. Strikingly, all stages of angiogenesis were decreased in ECs following exposure to *Olfml3^−/−^* microglia CM despite increased VEGF-A levels, suggesting that microglia-derived OLFML3 is a potent, VEGF-independent, pro-angiogenic factor in mouse brain ECs. 

The CM experiments in this study suggest that microglial OLFML3 is necessary for multiple parameters of microglia-mediated angiogenesis. We did observe mild variability in the effects of rhOLFML3 vs. microglial CM on EC differentiation. While the effects observed in EC viability and migration were consistent between experimental paradigms, CM from isogenic control microglia increased additional parameters of EC differentiation relative to rhOLFML3. This is consistent with the presence of additional pro-angiogenic factors in microglial CM. Indeed, we confirmed the expression of multiple pro-angiogenic factors in both isogenic control *Olfml3^−/−^* microglia, as well as secreted VEGF in microglia media. However, to our surprise, we observed a marked reduction in angiogenic parameters following EC exposure to *Olfml3^−/−^* microglia CM with a concurrent increase in pro-angiogenic molecules. At first glance, these findings appear contradictory. However, it is possible that secreted OLFML3 works synergistically with other pro-angiogenic molecules to increase EC angiogenesis. Thus, the effects of the loss of OLFML3 may represent not only the loss of a single potent angiogenic stimulus, but the absence of synergy between OLFML3 and another molecule(s). Therefore, the compensatory increase in VEGF-A was not sufficient to increase angiogenic parameters. 

OLFML3 has been implicated in the promotion of angiogenesis in several cancers. Our previous work has demonstrated that microglia-derived OLFML3 is also relevant in GBM, as it promotes a pro-tumorigenic microglial phenotype and glioma cell malignancy [21]. Here, we have identified a third pro-tumorigenic role for microglia-derived OLFML3: the promotion of angiogenesis. Importantly, this work suggests that microglia-derived OLFML3-mediated angiogenesis is VEGF-A independent. This may have significant implications for GBM patients. While anti-VEGF therapy targets EC survival, abolishes growth of tumor vessels and even prunes existing vessels [29], it generally does not affect pericyte numbers or function. Thus, pericytes and remaining basement membrane provide a platform for rapid revascularization. However, OLFML3 has been implicated in both EC-mediated neoangiogenesis and pericyte-mediated tumor revascularization. Tumor vasculature coverage by pericytes, cells which are necessary for EC survival [30], was reduced following anti-OLFML3 therapy in a lung carcinoma model. Looking ahead, it is possible that anti-OLFML3 therapy, administered concurrently with anti-VEGF therapy, may work synergistically in the GBM microenvironment to mitigate neoangiogenesis and tumor growth.

Microglia are increasingly recognized for their role in promoting angiogenesis in health and disease. Interestingly, hypoxia-preconditioned microglia potently induce angiogenesis in ECs derived from endothelioma (bEnd.3 ECs) through release of extracellular vesicles rich in TGFβ1 and up-regulation of the Smad2/3 pathway [31]. *Olfml3* is a direct target gene of the TGFβ1/Smad2, as mRNA levels increase ≥15-fold following TGFβ1 exposure [22]. Thus, it is tempting to consider the increased angiogenic effects of microglia-derived, TGFβ1-rich, extracellular vesicles observed in this study were the result of increased EC production of OLFML3. Therefore, it is possible that there are multiple mechanisms leading to microglial-mediated OLFML3-induced angiogenesis in health and cancer. 

We recognize limitations to the current study. Despite a marked reduction in *Olfml3* mRNA, we did not observe attenuation of angiogenic parameters in mouse brain microvascular ECs. This contrasts with the marked aberrations in vascular density and EC differentiation observed in mice heterozygous for *Olfml3* (*Olfml3^+/−^*) [18], which suggested that full expression of *Olfml3* is necessary for appropriate angiogenesis during development. In this present study, we utilized ECs from wildtype C57Bl/6 juvenile mice. The apparent discrepancy between these studies may be the result of genetic background differences or may indicate that the necessity of *Olfml3* in normal vascularization is temporally mediated. Moreover, multiple studies suggest that the pro-angiogenic effects of OLFML3 are mediated through both ECs and pericytes. Future experiments should explore the relationship between microglia-derived OLFML3, ECs and pericytes across genetic backgrounds.

In conclusion, we have demonstrated that exogenous OLFML3 promotes angiogenesis in primary brain microvascular ECs from C57Bl/6 mice. Moreover, OLFML3 is necessary to elicit maximal microglia-mediated EC angiogenesis. Considering our previous findings that microglia-derived OLFML3 promotes glioma cell malignancy and a pro-tumorigenic microglia phenotype, these results are particularly exciting. Taken together, our in vitro data suggest that microglia-derived OLFML3 may contribute to GBM progression through several mechanisms. 

## 4. Materials and Methods

### 4.1. Cell Culture and Reagents

C57BL/6 mouse primary brain microvascular endothelial cells (ECs) were purchased from CellBiologics, Inc. (C57-6023; Chicago, IL, USA). Cells were maintained in proprietary complete EC medium containing 2% fetal bovine serum (FBS), 0.1% VEGF, 0.1% epidermal growth factor, and 1% L-glutamine (CellBiologics, Chicago, IL, USA). Penicillin and Streptomycin (1%; Gibco™, ThermoFisher Scientific, Waltham, MA, USA) were added to culture medium. ECs were used between passages 3 and 6 and within one month after thawing. The N9 microglial cell line [32] was generously donated from Jyoti Watters at the University of Wisconsin School of Veterinary Medicine and has been authenticated by ATCC. N9 cells were used to generate *Olfml3* null (*Olfml3^−/−^*) and isogenic control cell lines using CRISPR/Cas9, as previously described [21]. N9 isogenic control and *Olfml3^−/−^* cells were maintained in DMEM (Gibco™, ThermoFisher Scientific, Waltham, MA, USA) supplemented with 10% FBS. All cells were confirmed to be Mycoplasma-free and maintained at 37 °C in a humidified incubator with 5% CO_2_. 

For all microglia CM experiments, N9 isogenic control and *Olfml3^−/−^* cells were grown to 90% confluency. CM was harvested and centrifuged at 200× *g* to remove any cellular debris. ECs were exposed to CM for 48 h at 37 °C in a humidified incubator with 5% CO_2_. For vehicle-controlled experiments, N9 microglial media were added to EC cultures.

For all co-culture experiments, ECs or microglia (2.5 × 10^4^) were seeded into 6-well plates. The alternate cell types (ECs or microglia; (1.5 × 10^4^)) were seeded into 0.4µm pore cell culture inserts (Nunc, ThermoFisher Scientific, Waltham, MA, USA). Cells were co-cultured for 48 h, followed by RNA isolation or acquisition of CM from the cell type seeded onto the plate.

### 4.2. Small Interfering RNA Targeting Olfml3 (Olfml3-siRNA) 

*Olfml3*-siRNA was designed and generated through ON-TARGETplus siRNA (Horizon Discovery, Boulder, CO, USA). Primary mouse brain microvascular ECs were seeded (8 × 10^4^) and grown to 70% confluency. ECs were transfected with 5µM *Olfml3*-targeted or scrambled siRNA and 6µL Dharmafect 4 transfection reagent (Horizon Discovery, Boulder, CO, USA). Cells were rested for 24 h following transfection, then assayed for *Olfml3* mRNA levels and used for experimentation. 

### 4.3. Human Recombinant OLFML3 Protein Generation

Human recombinant OLFML3 (rhOLFML3) was generated in our laboratory as previously described [21]. Briefly, the protein sequence for OFLML3, consisting of 406 amino acids, is 94.3% identical between human and mouse, as determined by a protein BLAST through the National Center for Biotechology Information. The OLFML3 sequence was cloned into pTXB1 Vector (NEB, N6707S) using *Olfml3* cDNA (Addgene MA, USA) as a template with the following primers: Forward—5′-GGTGGTCATATGGGGCCCAGCACCCCT-3′, and reverse—5′-GGTGGTTGCTCTTCCGCAAACCTCCTCCTCTTTCTTCCTCAT-3′. pTXB1-OLFML3 vector was electroporated into ClearColi^®^ BL21 (DE3) Electrocompetent cells (Lucigen). These cells have a genetically modified Lipopolysaccharide (LPS) that does not trigger endotoxic response in subsequent assays. Briefly, pTXB1-OLFML3 expressing ClearColi cells were induced (500 μM Isopropyl β-d-1-thiogalactopyranoside (IPTG)) at OD600 = 0.72 and incubated at 16 °C for 18 h. Cells were pelleted, lysed and incubated with Chitin resin. After washing, rhOLFML3 was stripped from Chitin beads using 50 mM Dithiothreitol (DTT) at 4 °C for 72 h. rhOLFML3 was eluted and concentrated using Pierce™ Protein Concentrator PES column, with 10,000 Da molecular weight cutoff (ThermoFisher). rhOLFML3 protein was subjected to Fast Protein Liquid Chromatography (FPLC) using HIPREP 16/60 SEPHACRYL S-200 column to remove residual DTT before BCA quantification and subsequent use in all experiments. 

### 4.4. Western Blot Analysis for OLFML3 in Microglial Conditioned Media

N9 isogenic control and *Olfml3^−/−^* microglia were grown to confluency under serum starvation conditions (0.1% FBS) in 150 mm cell culture plates. Media were collected and centrifuged at 150× *g* to pellet cells and cellular debris. Media were then passed through a <100 kD PES protein concentrator column (Thermo), centrifuged through a <3 kD PES spin column and concentrated to a volume of 250 µL. The concentrated media were precipitated using 50% saturated ammonium sulfate ((NH_4_)_2_SO_4_) for 72 h at −20 °C. Samples were thawed and centrifuged at 16,000× *g* for 20 min at 4 °C and resultant protein pellet was lysed using RIPA buffer. Equal micrograms of protein were loaded, electrophoresed on 15% SDS-PAGE gels and transferred to nitrocellulose membranes overnight at 4 °C. After blocking with 5% non-fat dry milk, membranes were incubated overnight at 4 °C in primary antibody solution containing a rabbit polyclonal anti-OLFML3 antibody generated by our laboratory in collaboration with Cocalico Biologicals (Reamstown, PA, USA), as previously described [21]. After washing with TBS-T, horseradish peroxidase secondary antibodies were applied for 1 h at room temperature and chemiluminescence images were acquired.

### 4.5. Cell Viability

Cell viability was performed at 48-h post-treatment paradigm. The Cell Titer Glo^®^ 2.0 Assay (Promega Corporation, Madison, WI, USA) was used according to the manufacturer’s protocol. Cells (2.5 × 10^4^) were seeded in 96-well black-sided plates. Titer Glo^®^ reagent was added to each well and the plate was incubated for 10 min at RT on a plate shaker, followed by luminescence recording via plate reader (BioTek800TS). Optical densities were recorded for 6 replicates per condition and the average optical density of media alone (blank) was subtracted from all experimental conditions. Three independent experiments were performed. 

### 4.6. Transwell Migration Assays

The modified Boyden chamber assay was used for analysis of cell migration. ECs (1.0 × 10^5^) suspended in complete EC media were seeded into 24-well plate with 8 µm pore polycarbonate filter inserts. Complete EC media were used in both the top insert and the receiver wells. Following 24-h incubation (37 °C, 5% CO_2_), inserts were removed, and the top of each insert was swabbed to remove non-migrated cells. Remaining cells attached to the bottom of the insert were fixed using 4% paraformaldehyde. The membranes were excised from the inserts and mounted to microscope slides using mounting medium containing 4′,6-diamidino-2-phenylindole (DAPI). Nine photographs were taken per membrane, with three technical replicates per experiment, using a brightfield microscope (Leica DM5000 B). Cells were identified by positive DAPI immunoreactivity and quantified via an ImageJ custom macro. Three independent experiments were performed. 

### 4.7. Tube Formation Assay

Differentiation was assessed via the tube formation assay. ECs (1.2 × 10^5^) were seeded onto reduced growth factor Matrigel (Corning, Inc., Corning, NY, USA) coated 24-well plates and incubated (37 °C, 5% CO_2_) for six hours. Based on pilot studies, we have previously determined that incubation for six hours produces robust, reproducible EC differentiation. Following incubation, images were acquired using a Leica EC4 camera mounted on a Nikon TMS inverted microscope. Each well was divided into a grid and 9 images were acquired from each well. Images were uploaded and analyzed using WimTube algorithm (Wimasis Image Analysis, Cordoba, Spain). Metrics of differentiation analyzed included: (1) total tube coverage: a sum of the length, in pixels, of all tubular structures in the image normalized to control conditions; (2) mean tube length: the average length, in pixels, of the tubular structure between branching points normalized to control conditions; (3) total loops: summation of the number of complete closure of the tubular structures; and (4) branching points: summation of the number of points where ≥3 tubes converge. Each condition was performed in triplicate per experiment. Three independent experiments were performed. 

### 4.8. Quantitative Real-Time PCR

Cells were pelleted and RNA was isolated using the Direct-zol MiniPrep kit (Zymo Research, Irvine, CA, USA) according to manufacturer’s specifications. Using one microgram of purified DNase treated RNA, cDNA was reverse transcribed using the High-Capacity cDNA Reverse Transcription Kit (Thermo Fisher—Applied Biosystems, ThermoFisher Scientific, Waltham, MA, USA). Primer sets were designed using NCBI primer design (https://www.ncbi.nlm.nih.gov/tools/primer-blast/index.cgi), accessed on 1 October 2021, and purchased through IDT (Table 1). Primer validation was performed using a 4× cDNA serial dilution series from untreated murine Ecs and microglia as template. The efficiency and fit of the generated curves were evaluated; primer sets that did not produce efficiency of at least 0.9 and R^2^ value of 0.95 from the cDNA dilution series were rejected. Only experimental quantification cycle (Cq) values that fell within the boundaries of the validated curves were used for analysis.

Triplicate 20 µL reactions using 50 ng cDNA template and 2X SsoAdvanced Universal SYBR Green Superix (Bio-Rad, Herules, CA, USA), as per manufacturer’s protocol, were run on a CFXConnect (Bio-Rad) machine, as previously described [33]. The average Cq was used as the data point for a given sample. mRNA expression values were quantified by the 2^−ΔΔCt^ method, whereby ΔCT = 18S Ct—gene of interest Ct.

### 4.9. Cell Culture Supernatant Measurement of VEGF-A

We measured microglial supernatant concentrations of VEGF-A with a commercially available ELISA kit, according to manufacturer’s instructions. (RayBio^®^ Mouse VEGF-A ELISA Kit, Code ELM-VEGF-1, RayBiotech Life, Peachtree Coreners, GA, USA). Supernatant was collected and immediately frozen at −80 °C until analysis. Samples underwent a single freeze–thaw cycle. Samples were plated without dilution; standards and samples were evaluated in triplicate. VEGF-A concentration was normalized to isogenic control concentration in each experiment.

### 4.10. Statistics

Statistical analysis was performed with Prism GraphPad V9.0.2 software (GraphPad by Dotmatics, San Diego, CA, 92108). Data were tested for normality via Shapiro-Wilks test. Data are presented as the mean ± SEM. Cell culture experiments were performed in technical triplicates, with three independent experimental replicates. Statistical significance was assessed via unpaired two-tailed Student’s *t*-test or ANOVA, with Tukey’s multiple comparisons test. Results were regarded as statistically significant for *p* < 0.05.

## Figures and Tables

**Table 1:** Primer sequences used for qPCR.

Gene	Sequence-F (5′ to 3′)	Sequence-R (5′ to 3′)
*Angpt2*	CCTCGACTACGACGACTCAGT	TCTGCACCACATTCTGTTGGA
*Fgf1*	CCTGACCGAGAGGTTCAAC	GTCCCTTGTCCCATCCACG
*Fgf2*	GCGACCCACACGTCAAACTA	TCCCTTGATAGACACAACTCCTC
*Hgf*	ATGTGGGGGACCAAACTTCTG	GGATGGCGACATGAAGCAG
*Vegfa*	GCACATAGAGAGAATGAGCTTCC	CTCCGCTCTGAACAAGGCT
*Vegfb*	AGCAGGTTTTGAAGTTCACCC	GGAGTGGGATGGATGATGTCAG
*Vegfc*	GAGGTCAAGGCTTTTGAAGGC	CTGTCCTGGTATTGAGGGTGG
*Vegfd*	TGAGCGATCATCCCGGTC	GCGTGAGTCCATACTGGCAAG

## Data Availability

All data is shared within the contents of this manuscript.
